# Case report: Teflon granuloma following microvascular decompression manifesting as light-triggered trigeminal neuralgia

**DOI:** 10.3389/fsurg.2022.904434

**Published:** 2022-07-22

**Authors:** Mustaqim Prasetya, Peter Adidharma, Takuro Inoue, Adi Sulistyanto, Fadhil, Selfy Oswari, Ryan Rhiveldi Keswani, Muhammad Kusdiansah, Yunus Kuntawi Aji, Abrar Arham

**Affiliations:** ^1^ Department of Neurosurgery, National Brain Center Hospital Prof. Dr. Dr. Mahar Mardjono Jakarta, East Jakarta, Indonesia; ^2^ Department of Neurosurgery, Subarukai Koto Memorial Hospital, Shiga, Japan

**Keywords:** trigeminal neuralgia, recurrent trigeminal neuralgia, light-triggered pain, microvascular decompression, Teflon granuloma

## Abstract

Trigeminal Neuralgia is commonly triggered by stimuli in the area of the trigeminal nerve innervation. We report an exceptionally rare case of a 61-year-old woman who complained of recurrent trigeminal neuralgia, which sole trigger was seeing a bright light. Teflon felt that was placed on the nerve root in the initial surgery was suspected of causing this rare type of trigeminal neuralgia. A reflex circuit linking luminance to trigeminal nerve activity may be implicated in activating a trigeminal nociceptive pathway by a bright light trigger.

## Introduction

Trigeminal neuralgia (TN) is a neuropathic facial pain characterized by sudden unilateral shock-like pain within one or more trigeminal nerve territories. Neurovascular compression is found in most patients with TN, but its pathogenesis is still controversial. Several hypotheses exist, such as peripheral cause, central cause, and peripheral origin central pathogeneses theories (1). The pain of TN itself is usually triggered by a stimulus on the trigeminal nerve territories, such as shaving, brushing teeth, washing, talking, smiling, drinking, or even soft touch (2).

Pain in the trigeminal nerve territory, such as ocular pain caused by bright light, is an established physiological phenomenon, suggesting the existence of a reflex circuit linking luminance to trigeminal nerve activity. A recent study revealed that bright light activates the trigeminal nociceptive pathway in experimental animals (3). However, a clinical case of trigeminal pain, in this instance TN, triggered by luminance to the trigeminal pathway has not been reported. We present a rare case of a TN patient whose sole trigger is exposure to bright light.

## Case presentation

A 61-year-old woman was presented to our clinic due to uncontrolled recurrent TN. Previously, she had a history of typical TN in the maxillary-mandibular division on the left trigeminal nerve territory for five years, triggered by light touch while brushing her teeth. The patient underwent microvascular decompression surgery (MVD) outside our centre three years before presentation, where neurovascular compression caused by the superior cerebellar artery was decompressed by inserting a shredded Teflon between the nerve root and the offending vessel. Following the initial MVD, she became pain-free without medication for a year. After that, the pain recurred, and she was referred to our clinic two years later.

**Figure 1:**
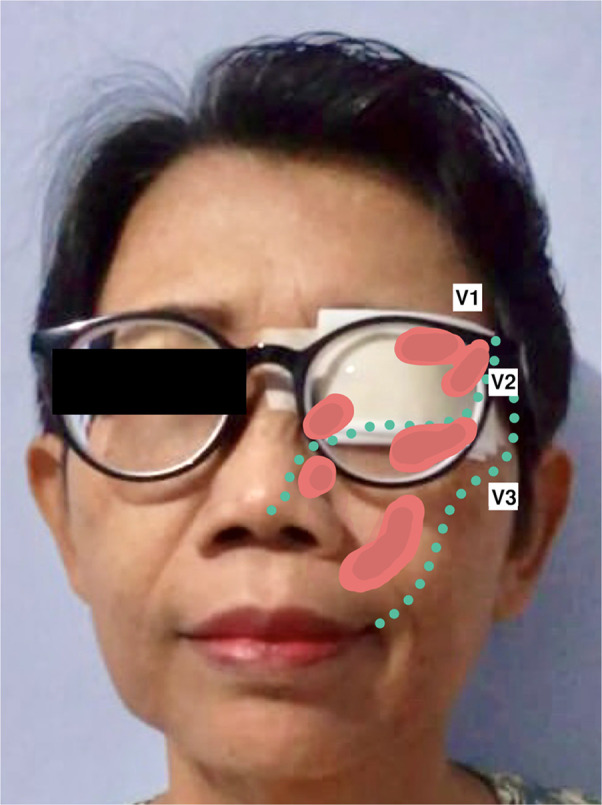
The patient had been using an eye patch to prevent episodes of recurrent trigeminal pain triggered by bright light, as illustrated in the picture above. Area of recurred pain are coloured in red (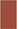), corresponding to the ophthalmic and maxillary branches of the trigeminal nerve; abbreviations: V1, ophthalmic branch of the trigeminal nerve; V2, maxillary branch of the trigeminal nerve; V3, mandibular branch of the trigeminal nerve.

**Figure 2:**
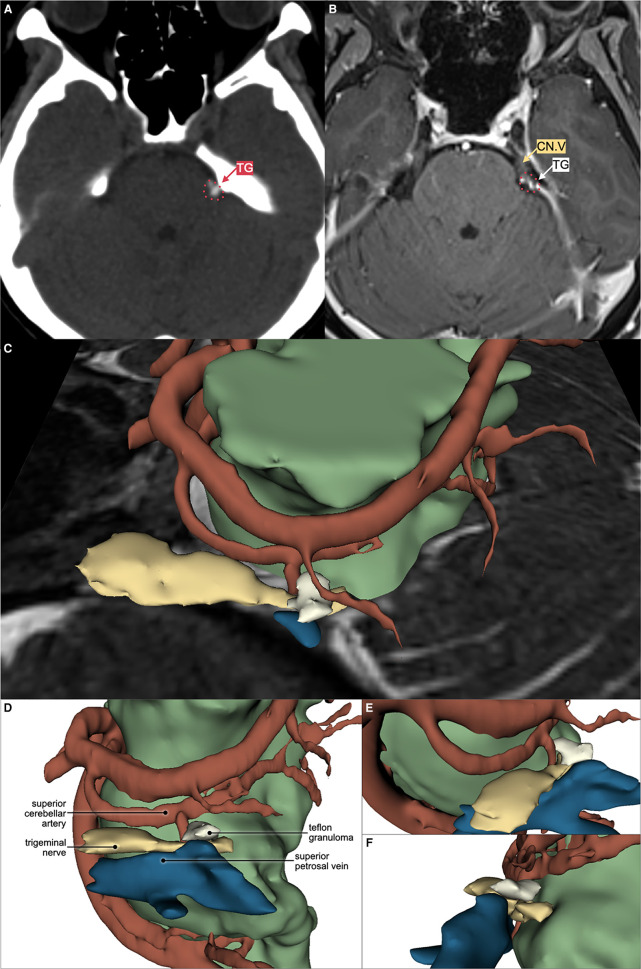
Axial computed tomography (CT) scan without contrast showing hyperdense lesion (**A**) and magnetic resonance imaging (MRI) T1 with gadolinium contrast (**B**) showing T1 low to medium intensity, contrast-enhancing lesion compressing the superolateral portion of left trigeminal nerve root entry zone (CN.V), suggesting Teflon granuloma (TG). Left superior (**C**), left lateral (**D**), left anterolateral (**E**), and posterolateral (**F**) view of the 3D reconstruction using 3D Slicer™ software showing the relation between Teflon granuloma, trigeminal nerve, superior cerebellar artery, superior petrosal vein, and the remaining brainstem parenchyma. Trigeminal nerve is coloured as yellow (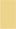); artery as red (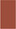); vein as blue (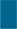); and brainstem parenchyma as green (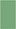).

Her recurrent facial pain was uniquely provoked by bright light, an exceptionally rare trigger for TN. Facial or oral stimuli did not trigger the pain. The pain exhibited typical electric shock-like pain similar to the initial presentation. However, it involved the left ophthalmic-maxillary branches of the trigeminal nerve and was accompanied by concomitant-continuous pain with throbbing sensation in the same territory, which was not present in her first pain episode. The severity was as follows: numeric rating scale (NRS) 10 and verbal rating scale (VRS) severe for paroxysmal pain; NRS 2 and VRS mild for the concomitant-continuous pain. The pain never lasted more than two minutes as she closed her eyes whenever a paroxysmal pain episode happened. Subsequently, she covered her left eye with a patch to avoid triggering the pain episodes (Figure 1). No autonomic symptoms were documented. The patient has been under medication of carbamazepine (200 mg BID) and gabapentin (300 mg BID); however, adequate pain control was not achieved (Barrow Neurological Institute; BNI pain intensity score: IV). General, neurological, and neuro-ophthalmological examinations were unremarkable. Magnetic resonance imaging (MRI) and pre-operative 3D reconstruction suggested a Teflon granuloma (TG) compressing the left trigeminal nerve root (Figure 2).

**Figure 3:**
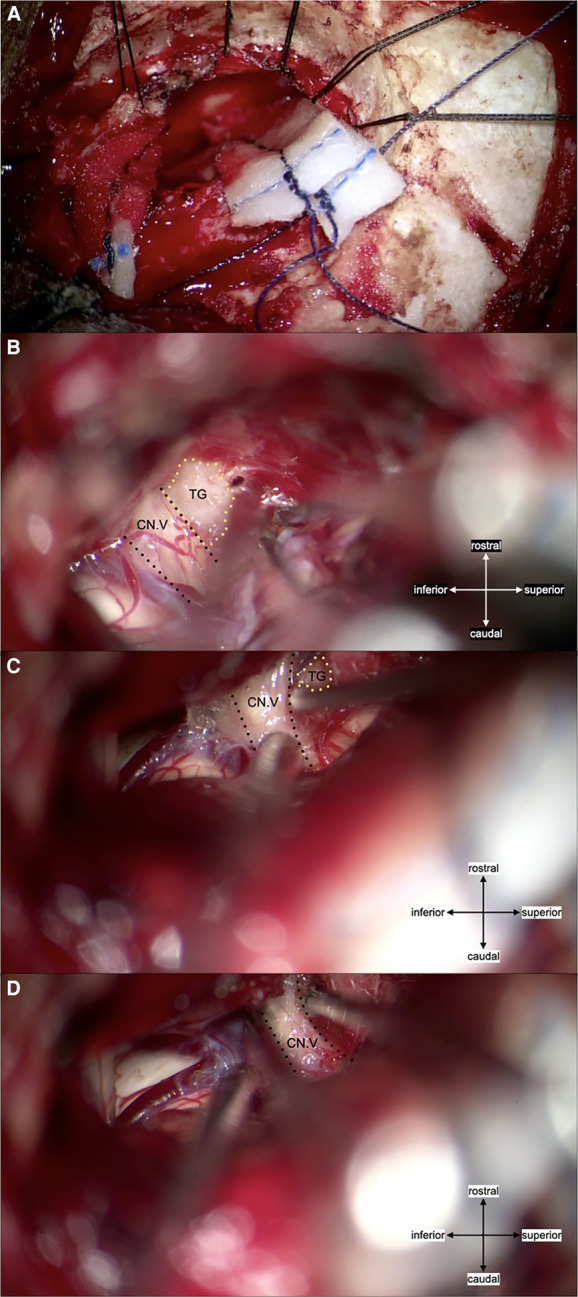
(**A**) craniotomy was conducted using the left retrosigmoid approach and the same skin incision as for the initial surgery. (**B**) Intraoperative findings showed a non-neurovascular origin compression (Teflon granuloma) of the trigeminal nerve root entry zone rostro-lateral portion. The granuloma was tightly adhered to the mildly atrophic trigeminal nerve, with no marked angulation. Local thickening of the arachnoid membrane was also present, adherent to the nerve. (**C**) The granuloma was debulked, and the remaining granulation was transposed by dissecting it free from the nerve root. Surgicel™ was used to avoid direct contact between the remaining TG and surrounding tissue. (**D**) Partial internal rhizotomy was conducted following the MVD; abbreviations: CN. V, trigeminal nerve; TG, Teflon granuloma.

A peripheral nerve block and prolotherapy procedure (supraorbital, supratrochlear, and infra-trochlear nerves with 2% lidocaine and 5% dextrose solution) were temporarily effective in reducing light-triggered paroxysmal pain for up to 24 h. A redo MVD was considered due to uncontrolled facial pain by medication. The second MVD was performed through the same skin incision and craniotomy (Figure 3). Intraoperatively, a TG was found compressing the rostro-lateral portion of the left trigeminal nerve root with severe adhesion. It was partially debulked until detachment from the root surface was achieved, then transposed away from the nerve root. A partial internal rhizotomy was conducted to ensure desirable pain control. She became pain-free immediately after surgery. Trigeminal deficits, such as facial numbness, dry eye, and masseter weakness, were absent. No neurological complications or other surgical complications occurred. The patient maintained a pain-free state without medication for a follow-up period of one year at our institution.

## Discussion

**Figure 4.**
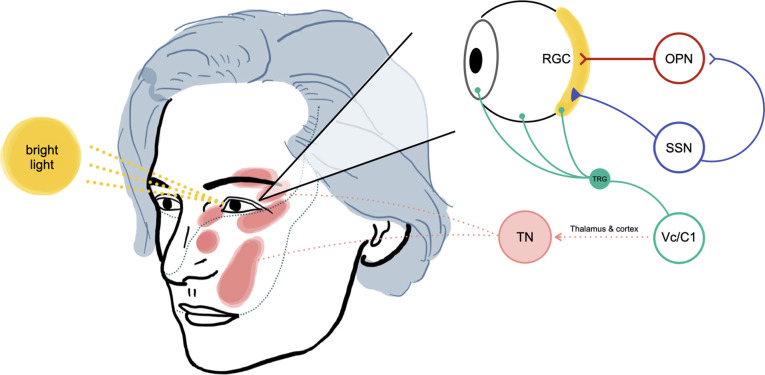
The proposed mechanism of light-triggered trigeminal neuralgia in our patient (adapted from Okamoto et al. experimental study) (3). The bright light stimulus will be received by retinal ganglion cells (RGC), then activate signaling into the olivary pretectal nucleus (OPN). The superior salivatory nucleus (SSN) will be activated, increasing parasympathetic outflow to the eye, causing vasodilation of ocular blood vessels and activation of ocular trigeminal afferent. Neurotransmitters will then be released due to these changes and passed to the trigeminal root ganglion (TRG) and Vc/C1 neurons (nociceptive neurons in the superficial laminae of the spinomedullary junction). Higher pain interpretation processes at the thalamus and cerebral cortex will also contribute to our patient's pathogenesis of light-triggered trigeminal neuralgia (TN).

The causes of TN include neurovascular compression, multiple sclerosis, tumours, diabetes mellitus, herpes simplex virus, allergy, and brain stem infarction. However, the pathogenesis behind it has been controversial and debated for a long time. Demyelination of the nerve root can be a source of ectopic impulses, leading to an ephaptic transmission that generates trigeminal pain (1). However, this peripheral theory cannot account for immediate electrophysiologic recovery and pain relief after MVD because transposition of the offending pathology can not achieve immediate remyelination. Another possible explanation is the central theory. Black et al. demonstrated that the trigeminal nucleus caudalis might have a pivotal role in generating TN in an experimental study (4). Kugelberg et al. supported the central theory by showing the long summation times, the tendency of the attack to be self-maintained, the long-lasting refractory period after an attack, and the efficacy of the antiepileptic drugs, such as carbamazepine. They speculated that the mechanism is situated centrally, probably in the spinal trigeminal nucleus (5). Several authors hypothesized that both peripheral and central processes were involved in TN as one theory alone can not explain the disease pathology rationally (6). Bederson et al. suggested that the central pain-gating mechanism could be diminished by demyelination of the trigeminal nerve root, which is commonly caused by vascular compression (7). Thus, the pathogenesis of TN has not been determined yet, and the trigeminal spinal nucleus, especially trigeminal subnucleus caudalis, is likely to be involved in the occurrence of TN. Okamoto et al. identified an intraocular neural circuit necessary for bright light to excite trigeminal nociceptive neurons in superficial laminae of trigeminal subnucleus caudalis (Vc/C1) (Figure 4). They demonstrated that the trigeminal neurons in superficial laminae at the Vc/C1 region encode luminosity. The superior salivatory nucleus is critical for light-evoked responses by Vc/C1 neurons which requires input through the trigeminal ganglion. Their report demonstrated that the pathogenetic area of TN in the brain stem is linked to luminance stimuli, suggesting light-induced TN may occur, as presented in this report (3, 8).

Our patient exhibited a different type of TN after her recurrence. The affected division changed to ophthalmic-maxillary branches after the recurrence from maxillary-mandibular branches in the initial symptom. The type of pain also altered to Type 2 TN, accompanied by concomitant continuous pain, whereas the initial symptom was typical (Type 1 TN). This discrepancy may be related to the different objects contacting the nerve root. In the initial surgery, the superior cerebellar artery was the offender, but a TG was a possible cause of recurrence, which was found in the second MVD. This observation supports the peripheral origin central pathogenesis theory in which the peripheral trigeminal nerve root could modulate the central pain-gating mechanism (7). Different input through the trigeminal ganglion may lead to various light-evoked responses by Vc/C1 neurons in the superior salivatory nucleus, which may explain the discrepancy in pain type between the initial and recurrent TN in this patient (3). Chen et al. reported an inflammatory response by a Teflon felt (9). Teflon contact on the nerve root may provoke inflammatory changes, a different pathology from the usual vascular compression. Our case indicates that different types of peripheral pathology may affect the central pathogenesis of TN in the trigeminal nucleus.

Our case is an example of iatrogenic recurrent TN caused by the inserted Teflon in the previous MVD. Relieving the compression by TG resulted in immediate and long-term pain control. Teflon felt is a commonly used implant in MVD surgery as it is durable, inert, and easy to manipulate. Inserting a shredded Teflon between the nerve root and the offending pathology is attainable and may cure the trigeminal pain. However, granulation tissue may form surrounding the Teflon and cause recurrent TN (9, 10). Sindou et al. demonstrated the importance of a non-compressive MVD technique to avoid recurrence in a large TN cohort (11). It is crucial that a Teflon felt should be placed away from the nerve root to avoid future adhesion and granulation formation (12).

## Conclusion

We presented a rare case of light-triggered trigeminal neuralgia. A Teflon granuloma may cause a unique and unusual type of neural modulation in the luminance to trigeminal nociceptive pathway, causing light-induced trigeminal neuralgia.

## Data Availability

The original contributions presented in the study are included in the article/Suplementary Material, further inquiries can be directed to the corresponding author/s.
